# Reduced functional connectivity between bilateral precuneus and contralateral parahippocampus in schizotypal personality disorder

**DOI:** 10.1186/s12888-016-1146-5

**Published:** 2017-02-02

**Authors:** Yikang Zhu, Yunxiang Tang, Tianhong Zhang, Hui Li, Yingying Tang, Chunbo Li, Xingguang Luo, Yongguang He, Zheng Lu, Jijun Wang

**Affiliations:** 1 Shanghai Key Laboratory of Psychotic Disorders, Shanghai Mental Health Center, Shanghai Jiao Tong University School of Medicine, South Wan Ping Road 600, Shanghai, 200030 People’s Republic of China; 2 Klinik und Poliklinik für Psychiatrie und Psychotherapie, Klinikum rechts der Isar, TU München, Munich, Germany; 3 Department of Medical Psychology, Faculty of Psychology and Mental Health, Second Military Medical University, Shanghai, People’s Republic of China; 4 Department of Psychiatry, Yale University School of Medicine, New Haven, CT 06516 USA; 5 Department of Psychiatry, Shanghai Tongji Hospital, Tongji University School of Medicine, 389 Xin Cun Road, Shanghai, 200065 People’s Republic of China; 6 Bio-X Institutes, Key Laboratory for the Genetics of Developmental and Neuropsychiatric Disorders, Ministry of Education, Shanghai Jiao Tong University, Shanghai, People’s Republic of China

**Keywords:** Schizotypal personality disorder, Functional connectivity, Resting state, Precuneus, Parahippocampus

## Abstract

**Background:**

Schizotypal personality disorder (SPD) is linked to schizophrenia in terms of shared genetics, biological markers and phenomenological characteristics. In the current study, we aimed to determine whether the previously reported altered functional connectivity (FC) with precuneus in patients with schizophrenia could be extended to individuals with SPD.

**Methods:**

Twenty subjects with SPD and 19 healthy controls were recruited from 4461 freshmen at a university in Shanghai and received a resting-state scan of MRI. All participants were evaluated by the Chinese version of Schizotypal Personality Questionnaire (SPQ) and the Chinese version of Symptom Checklist (SCL-90). The imaging data were analysed using the seed-based functional connectivity method.

**Results:**

Compared with the controls, SPD subjects exhibited reduced FC between bilateral precuneus and contralateral parahippocampus. In SPD group, SPQ total score was negatively correlated with FC between right precuneus and left parahippocampus (*r* = −0.603, *p* = 0.006); there was a negative trend between SPQ subscale score of suspiciousness and FC between left precuneus and right parahippocampus (*r* = −0.553, *p* = 0.014); and a positive trend was found between SPQ subscale score of odd or eccentric behaviour and FC between left precuneus and right superior temporal gyrus (*r* = 0.543, *p* = 0.016). As for the SCL-90 score, a similar negative trend was found between SCL-90 subscale score of suspiciousness and FC between right precuneus and left parahippocampus (*r* = −0.535, *p* = 0.018) in SPD group.

**Conclusions:**

Our findings suggest that the decreased functional connectivity between precuneus and contralateral parahippocampus might play a key role in the pathophysiology of schizophrenia spectrum disorder.

## Background

Schizotypal personality disorder (SPD) is categorized as a schizophrenia spectrum disorder in the DSM-5 [1]. It is linked to schizophrenia in terms of shared genetics, biological markers and phenomenological characteristics [2]. Although individuals with SPD experience less severe psychotic-like symptoms, they exhibit an array of cognitive dysfunctions that are qualitatively similar to those observed in schizophrenia, such as attention and information processing, working memory and executive functioning [3–7]. Thus studying individuals with SPD can be a powerful strategy for advancing knowledge regarding the pathophysiology of schizophrenia. Moreover, SPD individuals are often unmedicated and are rarely in acute episode, which offers an apparent advantage in avoiding such confounding factors.

Precuneus is increasingly obtaining attention from researchers in the neuroimaging studies of psychiatric disorders [8–14]. It is located in the medial area of the superior parietal cortex and is involved in episodic memory, visuospatial processing, reflections upon self and aspects of consciousness [15–17]. Converging evidence suggests that the precuneus is a functional core of the default network [18, 19]. The latest view is that there is a dynamic model of cross lifespan functional segregation and integration between precuneus-dorsal posterior cingulate cortex network and default network [20], which highlights the importance of this region in the neurodevelopment. Structural and functional abnormalities of the precuneus were frequently reported in many neurological and psychiatric conditions including Alzheimer’s disease and mild cognitive impairment [21–30], and depression [31–36]. In schizophrenia, several studies reported significant abnormalities in functional connectivity (FC) of precuneus. The study by Kraguljac et al. [37] found deficits in resting state functional connectivity between precuneus and hippocampus, and glutamate abnormalities in the hippocampus in unmedicated patients with schizophrenia. Although the authors did not observe a direct relationship between these abnormalities, their findings partly elucidated the brain structures of the functional connectivity alterations in schizophrenia. The study by Gong et al. [10] provided genetic evidence of functional connectivity of precuneus. They found that both precuneus and its functional connectivity could be linked with DISC1 polymorphisms, which plays a role in both neural signalling and neurodevelopment and have been associated with schizophrenia. In first episode schizophrenia, another study by Guo et al. [11] found that FC between precuneus and bilateral Heschl’s gyri was not only abnormal, but also its correlation with a cognitive component of event-related potentials (ERPs), P300 amplitude, was absent. Although no study reported FC of precuneus in SPD, Nenadic et al. [38] did found the positive correlation of right precuneus with negative schizotypy scores among 59 healthy non-clinical volunteers using voxel-based morphometry (VBM). Therefore, precuneus is one of key areas for us to understanding schizophrenia spectrum disorders.

In the present study, we explored functional connectivity across whole brain with precuneus as the seed region in undergraduate students with schizotypal personality traits. We hypothesized that precuneus FC would be altered in these SPD subjects and could be associated with their schizotypal traits or subclinical symptoms.

## Methods

### Participants

Twenty subjects with SPD and 19 HCs were recruited from 4461 freshmen at a university in Shanghai. All participants were evaluated by a 74-item scale of the Chinese version of Schizotypal Personality Questionnaire (SPQ) and a 90-item scale of the Chinese version of Symptom Checklist (SCL-90). The Schizotypal Personality Questionnaire (SPQ) is a popular method of measuring both normal variability and abnormal degrees of schizotypy [39]. It has 9 subscales, including ideas of reference, social anxiety, odd beliefs/magical thinking, unusual perceptual experiences, eccentric/odd behavior and appearance, no close friends, odd speech, constricted affect, suspiciousness/paranoid ideation. For schizotypy and SPQ, the three higher-order factors proposed are Cognitive Perceptual (Ideas of Reference/Suspiciousness, Magical Thinking, and Unusual Perceptions), Interpersonal (No Close Friends/Constricted Affect and Social Anxiety), and Disorganized (Eccentric Behavior and Odd Speech) [40]. The Symptom Checklist-90 (SCL-90) is a questionnaire that is widely applied self-assessment tool for individuals with a broad range of mental disorders and symptom intensity [41]. It contains 90 items, yielding nine scores for primary symptom dimensions and three for global distress. The symptom dimensions comprise somatization, obsessive–compulsive behavior, interpersonal sensitivity, depression, anxiety, hostility, phobic anxiety, paranoid ideation (Suspiciousness), and psychoticism. The main global index of distress is the global severity index (GSI), which is the average of all responses. The test-retest reliability of the Chinese version SPQ after 4 weeks was 0.84 [42]. We defined top 10% of SPQ total scores in our screened sample as high SPQ scores. These eligible subjects were further interviewed by senior psychiatrists with Structured Clinical Interview for DSM-IV Axis II (SCID-II), and all the subjects in the SPD group in the present study met DSM-IV diagnostic criteria for SPD. The control subjects of SPD were randomly selected from those participants with low 10% of SPQ total scores. Both SPD group and control group underwent brain magnetic resonance imaging (MRI), and their resting-state functional MRI (rs-fMRI) data was collected. Three of them were excluded because of the poor quality of MRI scanning, and whose background data were not include in this report. The study was approved by the local institutional ethics board (Institutional Review Board of Shanghai Mental Health Center, Shanghai Jiao Tong University School of Medicine) and carried out in accordance with the Declaration of Helsinki. Written informed consent was obtained from each participant prior to inclusion.

### MRI acquisition

All images were acquired on a 3.0-T MR scanner (Trio, Siemens, Erlangen, Germany). A high-resolution T1-weighted sequence with the following parameters was used: repetition time (TR) = 1900 ms, echo time (TE) = 2.46 ms, flip angle = 9°, field of view (FOV) = 256 × 256 mm2, slice thickness = 1 mm and 192 slices. The rs-fMRI data were acquired using a gradient echo-planar imaging pulse sequence with the following parameters: TR = 2000 ms, TE = 25 ms, flip angle = 90°, FOV = 240 × 240 mm2, voxel size = 3.446 × 3.446 × 5 mm3, slice gap = 0. Each brain volume comprised 32 axial slices. Participants were asked to lie down and remain motionless, keep eyes open and fixation on a cross on the screen during the scan.

### Image processing

The rs-fMRI preprocessing was carried out using Data Processing Assistant for Resting-State fMRI (DPARSF version 2.0, http://www.restfmri.net). After the first 10 volumes were discarded, the images were corrected for slice timing and head motion. The data with over 3° or 3 mm movement were excluded from further analysis. The functional images were normalized to standard Montreal Neurological Institute (MNI) space and resampled to 3 × 3 × 3 mm3. Following the spatial smooth with a 4-mm full width at half-maximum (FWHM) Gaussian kernel, data were temporally band-pass filtered (0.01–0.08 Hz) and linearly detrended. The final step of preprocessing was linear regression to remove several sources of spurious covariates, including the effect of the average signals from white matter region, cerebral spinal fluid (CSF) region and whole brain signals, and the six head-motion parameters. We used the Resting-state fMRI Data Analyze Toolkit (Rest version 1.6, http://www.restfmri.net) to calculate the functional connectivity (FC) with bilateral precuneus as region-of-interest (ROIs). The two ROIs of bilateral precuneus were identified by an AAL template. Through computing the Pearson correlation coefficients between the time course of ROI voxel and that of all other brain voxels, we obtained the results of cross-correlation FC analysis. This was followed by normalization to Z-scores from correlation coefficients for each voxel with Fisher’s transformation. Finally, we extracted the individual Z-score maps for the preparation of group analysis.

### Statistical analysis

We also used the Resting-state fMRI Data Analyze Toolkit (Rest version 1.6, http://www.restfmri.net) to perform the group analysis. An independent *t*-test analysis was conducted with a significant threshold of *p* < 0.001 at the voxel level and *p* < 0.05 at the cluster scale (corrected for multiple comparisons using Monte Carlo simulation with the program Alphasim in AFNI). Then, we extracted the average Z-scores from each significantly different FC cluster with bilateral precuneus between two groups to perform two-tailed Pearson correlation analysis with SPQ or SCL-90 scores. The level of significance was set to *p* < 0.01.

## Results

### Demographic and clinical assessment

The demographic and clinical assessment of control and SPD groups were shown in Table 1. No significant difference was found between two groups in age and gender. All participants were undergraduate students. The SPQ scores and SCL-90 score in SPD group were significantly higher than those in the control group (*p* < 0.001).

**Table 1:** Demographic and clinical assessment

Variable (mean ± S.D.)	Control group	SPD group	*P*
	(*n* = 17)	(*n* = 19)	
Age (years)	19.71 ± 0.71	19.98 ± 0.82	0.305
Gender (M/F), n	16/1	17/2	-
SCL-90 total score	24.82 ± 22.18	114.42 ± 65.83	<0.001*
SPD total score	11.71 ± 4.09	47.42 ± 9.37	<0.001*
SPD subscale:			
1. Ideas of Reference	2.76 ± 1.60	7.11 ± 1.94	<0.001*
2. Social Anxiety	0.65 ± 0.93	5.58 ± 1.84	<0.001*
3. Odd Beliefs/Magical Thinking	1.29 ± 1.21	3.89 ± 1.76	<0.001*
4. Unusual Perceptual Experiences	1.12 ± 1.65	5.84 ± 2.63	<0.001*
5. Eccentric/Odd Behavior and Appearance	0.59 ± 0.71	4.26 ± 1.88	<0.001*
6. No Close Friends	0.94 ± 1.09	4.32 ± 2.21	<0.001*
7. Odd Speech	2.00 ± 1.00	6.95 ± 1.55	<0.001*
8. Constricted Affect	1.59 ± 2.00	4.42 ± 2.04	<0.001*
9. Suspiciousness/Paranoid Ideation	0.76 ± 0.83	5.05 ± 2.48	<0.001*

*S.D.* standard deviation, *M* male, *F* female, *SPQ* schizotypal personality questionnaire, *SPD* schizotypal personality disorder

*p < 0.05 significant two-sided testing

### Group differences in FC of bilateral precuneus

**Fig. 1:**
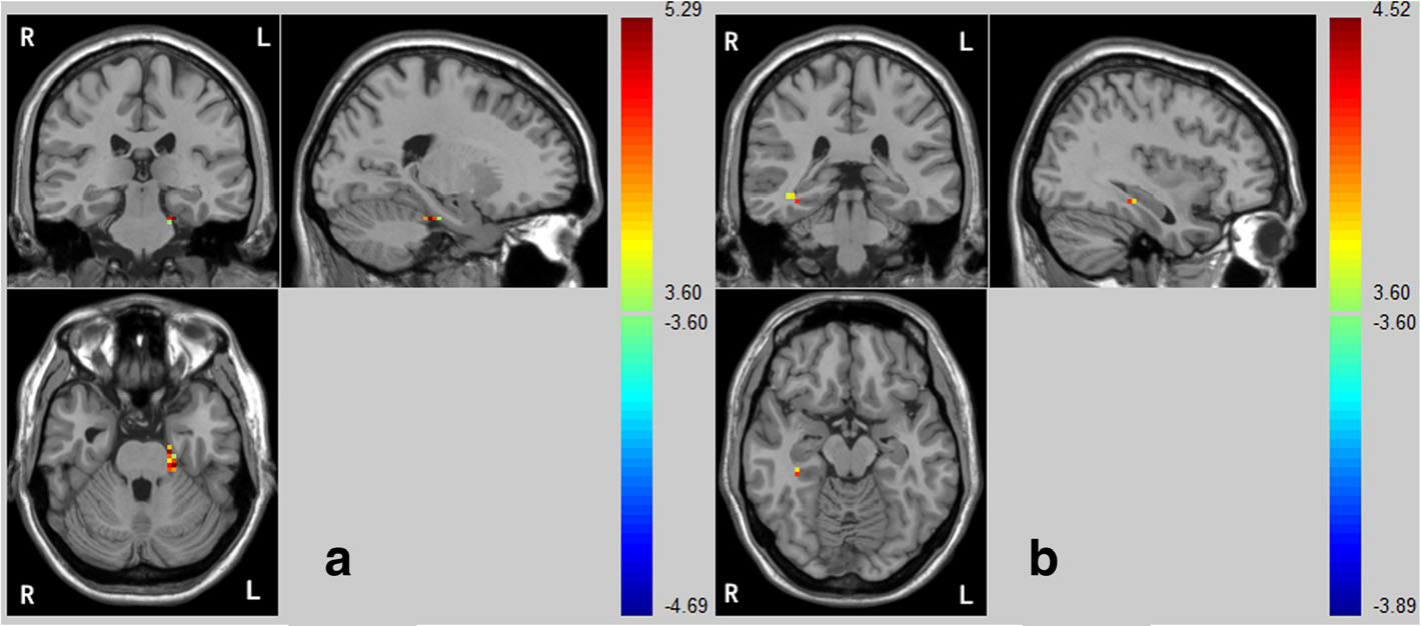
Compared with the control group, the SPD group exhibited decreased FC between the bilateral precuneus and opposite lateral (**a**: left; **b**: right) parahippocampus

Compared with control group, SPD group showed decreased FC between right precuneus and bilateral parahippocampus, decreased FC between right precuneus and right middle temporal gyrus, but increased FC between right precuneus and right middle frontal gyrus. As for left precuneus, SPD group only showed reduced FC with right parahippocampus and right superior temporal gyrus (see Table 2 and Fig. 1).

**Table 2:** Group differences in functional connectivity of bilateral precuneus

Direction	Region	Cluster size	Peak coordinates			SPD^a^ mean (sd)	HC^a^ mean (sd)
			x	y	z		
PCUN.R							
control > SPD	PHG.L	16	−21	−27	−27	−0.065 (0.092)	0.151 (0.101)
	PHG.R	9	39	−39	−12	−0.009 (0.131)	0.228 (0.143)
	MTG.R	10	69	−42	3	−0.194 (0.175)	0.074 (0.188)
control < SPD	MFG.R	28	39	39	33	0.217 (0.229)	−0.083 (0.122)
PCUN.L							
control > SPD	PHG.R	6	36	−33	−15	0.008 (0.138)	0.258 (0.178)
	STG.R	9	30	−57	27	−2.843 (0.181)	0.252 (0.174)

*SPD* schizotypal personality disorder, *PCUN.R* right precuneus, *PHG.L* left parahippocampus, *PHG.R* right parahippocampus, *MTG.R* right middle temporal gyrus, *MFG.R* right middle frontal gyrus, *PCUN.L* left precuneus, *PHG.R* right parahippocampus, *STG.R* right superior temporal gyrus

^a^voxel threshold *p* < 0.001, cluster threshold *p* < 0.05 using Alphasim correction

### Correlations of FC of precuneus with SPQ score and SCL-90 score

**Fig. 2:**
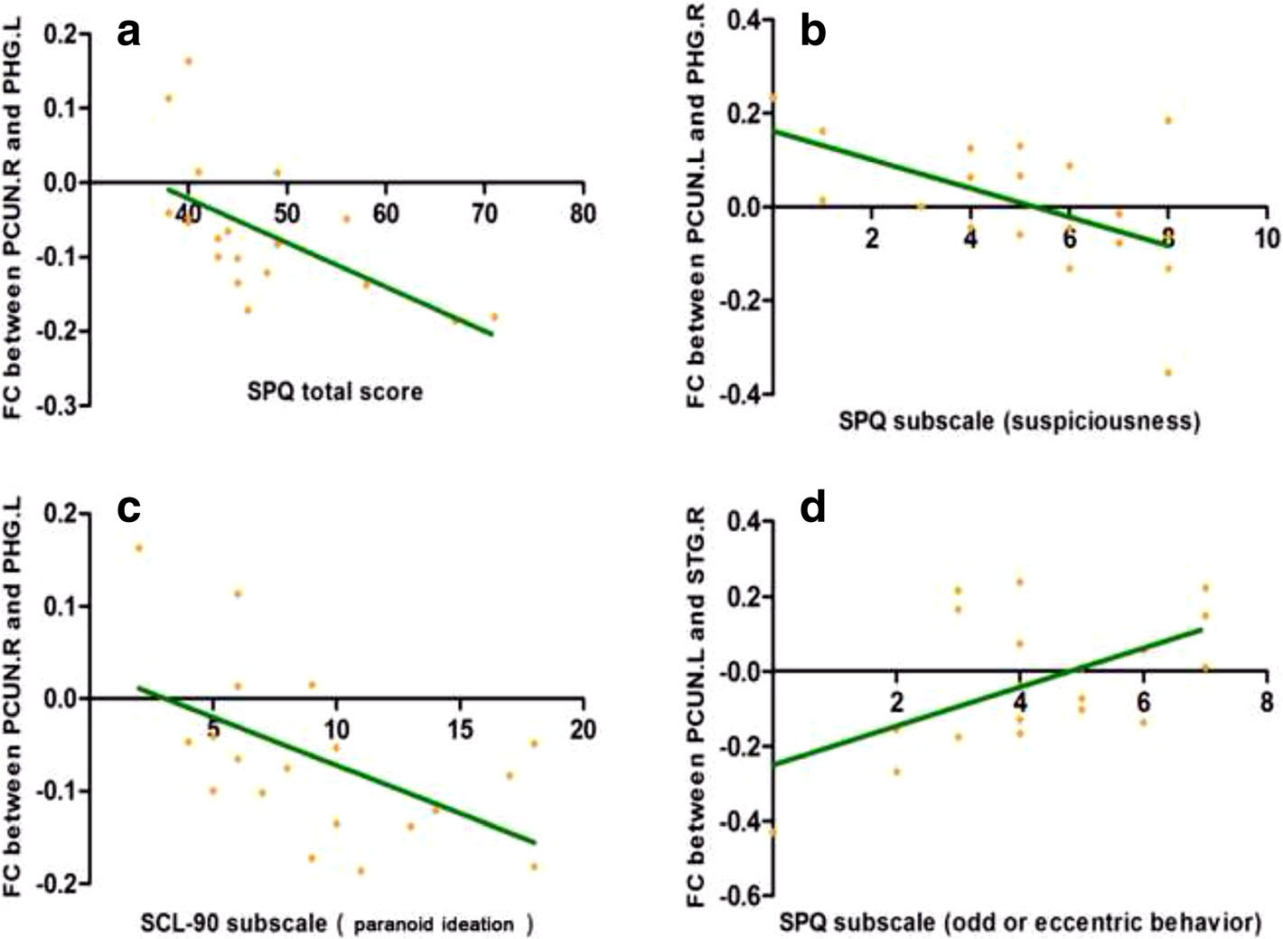
In SPD group, higher SPQ total score was found to be negatively associated with FC between the right precuneus and left parahippocampus (*r* = −0.603, *p* = 0.006) (**a**); there was a negative trend between SPQ subscore (suspiciousness) and FC between the left precuneus and right parahippocampus (*r* = −0.553, *p* = 0.014) (**b**); a similar negative trend was found between SCL-90 subscore (paranoid ideation) and FC between the right precuneus and left parahippocampus (*r* = −0.535, *p* = 0.018) (**c**); a positive trend was found between SPQ subscore (odd or eccentric behavior) and FC between left precuneus and right superior temporal gyrus (*r* = 0.543, *p* = 0.016) (**d**). Abbreviation: PCUN.R, right precuneus; PHG.L, left parahippocampus; PCUN.L, left precuneus; PHG.R, right parahippocampus; STG.R, right superior temporal gyrus; SPQ, Schizotypal Personality Questionnaire; SCL-90, Symptom Checklist

In SPD group, SPQ total score was found to be negatively correlated with FC between right precuneus and left parahippocampus (*r* = −0.603, *p* = 0.006); there was a negative trend between SPQ subscale score of suspiciousness and FC between left precuneus and right parahippocampus (*r* = −0.553, *p* = 0.014); and a positive trend was found between SPQ subscale score of odd or eccentric behaviour and FC between left precuneus and right superior temporal gyrus (*r* = 0.543, *p* = 0.016). As for the SCL-90 score, a similar negative trend was found between SCL-90 subscale score of paranoid ideation and FC between right precuneus and left parahippocampus (*r* = −0.535, *p* = 0.018) in SPD group (see Fig. 2). In control group, a positive correlation was found between SPQ subscale score of constricted affect and FC between right precuneus and right middle temporal gyrus (*r* = 0.670, *p* = 0.003).

## Discussion

This is the first study of FC of precuneus in SPD. In the present study, 20 SCID-II diagnosed SPD subjects from a pool of 4461 first year college students were compared with 19 subjects of low SPQ score and bilateral precuneus were selected as ROIs in functional connectivity analysis of resting state MRI scan. There were two main findings. First, FC of right precuneus with bilateral parahippocampus and right middle temporal gyrus, FC of left precuneus with right parahippocampus and right superior temporal gyrus, was reduced in SPD group as compared to the control group. There is an increased FC of right precuneus with right middle frontal gyrus in SPD subjects. Second, in the SPD group, individuals with higher SPQ total scores and SCL-90 subscale score of paranoid ideation showed stronger reduction in FC between right precuneus and left parahippocampus. These results indicate the fronto-parietal and temporal-parietal connection alterations in the unmedicated individuals with SPD and associations of hippocampal-parietal FC reduction with schizotypal traits.

The precuneus plays a pivotal role in a wide spectrum of highly integrated tasks, including visuo-spatial imagery, episodic memory retrieval and self-processing operations [15]. Furthermore, the precuneus is regarded as a hub in the functional connectome, also as part of the default mode network [18, 19]. As expected, we found that the altered FC of precuneus among the individuals with SPD. During the resting state, the control group showed reduced fronto-parietal and increased temporal-parietal connections, relative to the SPD group. One possible interpretation is that activity in the fronto-parietal control network (FPC) decrease and activity in the default mode network (DMN) increase. It has been well documented that the FPC-DMN anti-correlation is affected in schizophrenia patients [43–46]. The present study found a reduced FC between right precuneus and right middle temporal gyrus, but an increased FC between right precuneus and right middle frontal gyrus, supporting that the FPC-DMN anti-correlation is also affected among SPD subjects. Recently, Xin et al. [47] found that individual differences in empathic ability were significantly correlated with the FPC-DMN anti-correlation.

We further found that a reduced FC between right precuneus and left parahippocampus was significantly correlated with SPQ total scores and SCL-90 subscale score of paranoid ideation among SPD patients. Kraguljac et al. [37] reported hippocampal-parietal dysconnectivity in unmedicated patients with schizophrenia, and they demonstrated that the peak area of FC deficits between the hippocampus and precuneus in schizophrenic patients lies within the central area of the precuneus that is thought to be relevant for cognition. Our results are in consistency with these findings and demonstrate more prominent schizotypal traits are linked with more remarkable hippocampal-parietal dysconnectivity. Suspiciousness is categorized as cognitive perceptual disturbance according to the higher-order models for SPQ [40, 48]. It is also involved in the social cognition and metacognition, for instance, the evaluation and estimation on others according to common sense.

Moreover, we also found that a reduced FC between left precuneus and right parahippocampus was inversely correlated with SPQ subscale score of suspiciousness in SPD group. These results further supported the associations of FC between precuneus and parahippocampus with schizotypal traits. Previous studies supported that abnormal FC between precuneus and parahippocampus possibly plays a role in the continuum from individuals with schizotypal personality traits to developing schizophrenia. For instance, Allen et al. [49] recently reported that people at clinical high risk for psychosis showed parahippocampal hypoactivation during memory encoding. The authors also found that the parahippocampal response was correlated with the level of striatal dopamine function in the clinical high-risk individuals, which was absent in controls. Thus, they proposed that parahippocampal hypoactivation is related to an increased vulnerability to schizophrenia [49].

Several limitations should be considered while interpreting our results. First, the small sample size reduced detecting power, leading that the corrected clusters were relatively small. Second, our results only found a continuum of FC alteration in the individuals with schizotypal personality disorder and its direct validation in schizophrenia patients is still needed. Lastly, we did not detect FC of each subdivision in precuneus, which has been demonstrated to be involved in different functions [16, 17]. Therefore, we would like to keep our findings in the present study as preliminary.

## Conclusion

Taken together, our findings demonstrated that the functional connectivity between precuneus and parahippocampus, between precuneus and middle/superior temporal gyrus, might play a key role in the pathophysiology of schizophrenia spectrum disorder.

## Abbreviation

CSF: Cerebral spinal fluid
FC: Functional connectivity
FOV: Field of view
FWHM: Full width at half-maximum
MNI: Montreal neurological institute
MRI: Magnetic resonance imaging
ROIs: Region-of-interest
rs-fMRI: Resting-state functional MRI
SCID-II: Structured clinical interview for DSM-IV Axis II
SCL-90: Symptom checklist
SPD: Schizotypal personality disorder
SPQ: Schizotypal personality questionnaire
TE: Echo time
TR: Repetition time
